# Bone health in phenylketonuria: a systematic review and meta-analysis

**DOI:** 10.1186/s13023-015-0232-y

**Published:** 2015-02-15

**Authors:** Serwet Demirdas, Katie E Coakley, Peter H Bisschop, Carla E M Hollak, Annet M Bosch, Rani H Singh

**Affiliations:** Department of Paediatrics, Emma Children’s Hospital, Academic Medical Center, University of Amsterdam, Amsterdam, The Netherlands; Nutrition and Health Sciences and Molecules to Mankind Programs, Laney Graduate School and Department of Human Genetics, Emory University, Atlanta, GA USA; Department of Internal Medicine, Division of Endocrinology and Metabolism, Academic Medical Center, University of Amsterdam, Amsterdam, The Netherlands; Metabolic Nutrition and Genetics Program Department of Human Genetics, Emory University Atlanta GA United States, Atlanta, GA USA; Division of Metabolic Disorders, Emma Children’s Hospital, Academic Medical Centre, Meibergdreef 9, Amsterdam, AZ 1105 The Netherlands

**Keywords:** Phenylketonuria, PKU, Bone mineral density, BMD, Bone turnover markers, Phenylalanine, Osteopenia, Osteoporosis, Meta-analysis, Systematic review

## Abstract

Patients with Phenylketonuria (PKU) reportedly have decreased bone mineral density (BMD). The primary aim of this study was to perform a systematic review and meta-analysis to determine the extent and significance of low BMD in early treated patients with PKU. Secondary aims were to assess other bone status indicators including bone turnover markers (BTM) and to define areas for future research. Two research teams (Amsterdam, Netherlands and Atlanta, USA) performed literature searches for articles reporting data on BMD, osteopenia and osteoporosis, BTM or other bone indicators in patients with PKU. Included articles were compared between research teams and assessed for quality and risk of bias. A total of 13 unique articles were included; 11/13 articles reported BMD including a total of 360 patients. Ten out of 11 articles found BMD was significantly lower in patients with PKU. Meta-analyses for total BMD (TBMD; 3 studies; n = 133), lumbar spine BMD (LBMD; 7 studies; n = 247), and femoral neck BMD (FBMD; 2 studies; n = 78) Z-scores were performed. Overall effect sizes were: TBMD −0.45 (95% CI −0.61, −0.28); LBMD −0.70 (95% CI −0.82, −0.57); FBMD −0.96 (95% CI −1.42, −0.49). Definitions of osteopenia and osteoporosis were highly heterogeneous between studies and did not align with World Health Organization standards and the International Society for Clinical Densitometry positions on BMD measurement. Despite individual study findings of low BMD indicating higher risk of osteoporosis, pooled available data suggest reduction in BMD is not clinically important when using standard definitions of low BMD. Results from studies evaluating BTM are inconclusive. Phenylalanine concentration, vitamin D, PTH, and nutrient intake do not correlate with BMD or BTM. We recommend forthcoming studies use standard definitions of low BMD to determine clinical implications of BMD Z-scores below 0, explore cause of low BMD in the subset of patients with low BMD for chronological age (Z-score < −2) and assess fracture risk in patients with PKU.

**Electronic supplementary material:**

The online version of this article (doi:10.1186/s13023-015-0232-y) contains supplementary material, which is available to authorized users.

## Introduction

Phenylketonuria (PKU, ORPHA79254, MIM 261600) is a genetic disorder caused by mutations in the gene coding for phenylalanine hydroxylase (PAH; EC 1.14.16.1). As a consequence, the essential amino acid phenylalanine (Phe) cannot be converted to tyrosine and accumulates in the blood. Phe is transported across the blood–brain barrier and high concentrations can lead to mental retardation and behavioural and physical abnormalities. Implementation of newborn screening to detect PKU across the world since the 1960s has enabled early diagnosis and treatment. Early dietary treatment results in near normalization of outcomes for patients with the disorder [1].

The success of dietary treatment has, however, led to the discovery of secondary issues in the life-long treatment of PKU [1-8]. First reported in 1962, one of the complications seen in early and continuously treated patients is abnormal bone status [9]. Initially examined by radiological assessment, Feinberg et al. [9] described calcified spicules of cartilage projecting into the distal metaphyses of growing long bones in a sample of 33 patients with PKU ranging from infants to young adults. These findings were later supported by Murdoch et al. [10] and led to further studies assessing bone status in PKU by quantitative ultrasound (QUS) [11], peripheral quantitative computed tomography (pQCT) [12] and dual-energy X-ray absorptiometry (DXA) [13-17]. Low bone mineral density (BMD), an important risk factor for skeletal fractures, has since been reported by many studies [16,17].

A recent systematic review reported spine bone mineral density (BMD) was 0.100 g/cm^2^ lower (95% CI, −0.110, −0.090 g/cm^2^) in 67 subjects with PKU, compared to 161 controls collected from 3 studies [18]. This review, however, has methodological limitations: ascertainment bias by inclusion of late diagnosed patients who may suffer from cognitive delays and less physical activity potentially affecting the bone outcomes; lack of literature quality appraisal and assessment of bias; and no correction for age, gender and ethnicity on BMD data (based on g/cm^2^).

Most studies on bone in patients with PKU agree that bone is affected; however, there are significant gaps in knowledge and no consensus on the degree and implications of bone abnormalities, biological causes and risk-factors for low BMD [4,5,14,19,20], and the identification of subgroups of patients at-risk for fractures and compromised bone status [13,20,21]. To investigate these knowledge gaps, we combined the efforts of two international centers to perform a systematic review on bone status in PKU. Our primary aim was to systematically review the literature concerning bone status in early treated patients with PKU to perform a meta-analysis on BMD, corrected for bias, age and gender. Secondary aims were to assess other indicators of bone status including bone turnover markers (BTM) and to define areas for future research on bone status in PKU.

## Materials and methods

### Research question

Two centers for metabolic diseases in Atlanta, Georgia (USA) and Amsterdam (Netherlands) performed separate searches for literature concerning bone health in patients with PKU according to the Preferred Reporting Items for Systematic Reviews and Meta-Analyses (PRISMA) [22]. Both centers included similar research questions, review strategies and proposed outcomes, thus efforts were combined. Whereas the Atlanta research group focused on assessing the effects of nutrient intake, blood Phe concentration, and adjunctive therapy on bone status indicators (search 1); the group in Amsterdam focused on a comprehensive meta-analysis of BMD and an assessment of BTM in early diagnosed patients with PKU (search 2). The protocol for the Atlanta systematic review is registered with the ‘International prospective register of systematic reviews’ (PROSPERO) as systematic review number CRD42014009176 [23].

Inclusion criteria for search 1 were primary research or review articles, human research including subjects with PKU or hyperphenylalaninemia, and written in English only. Exclusion criteria were articles unrelated to PKU, animal studies, studies including in vitro results only, and studies that did not include BMD, bone mineral content (BMC), bone turnover, or measures of bone metabolism.

Inclusion criteria for search 2 were original studies (randomized controlled trial, cohort or case–control studies) of early diagnosed and treated patients with PKU studying either BMD or BTM with a quality rating of acceptable or better according to quality appraisal. Exclusion criteria were reviews (however reference lists were viewed for relevant articles), studies that include pregnant patients, articles published in a language other than English or Dutch and articles not meeting the inclusion criteria.

### Methodology

#### Databases

Literature eligible for inclusion in search 1 was retrieved from PubMed and EMBASE databases through a computerized search with assistance from a trained Emory University librarian. The initial search was performed in 2013, with an updated search completed in May 2014 to ensure the inclusion of recently published articles. As an example, we provide here the MEDLINE® search: (pku[All Fields] OR (“phenylketonurias” [MeSH Terms] OR “phenylketonurias” [All Fields] OR “phenylketonuria” [All Fields])) AND (“bone and bones” [MeSH Terms] OR (“bone” [All Fields] AND “bones” [All Fields]) OR “bone and bones”[All Fields] OR “bone” [All Fields]). All articles and abstracts retrieved through PubMed and EMBASE searches were downloaded in PDF format through Emory University open access or requested through Illiad, a document-delivery service, if full text was not available.

A computerized search with the help of a trained University of Amsterdam librarian in MEDLINE®, EMBASE and The Cochrane Library [24] was performed for search 2. The databases were searched initially in October 2013 and last in June 2014. No limits were used in the searches. As an example, we provide here the search used in MEDLINE®: (“Phenylketonurias” [mh] OR phenylketon* [tiab] OR “PKU” [tiab] OR hyperphenylalaninaemia[tiab] OR hyperphenylalaninemia [tiab]) AND ((minerals[mh] OR mineral*[tiab] OR “Bone Diseases, Metabolic” [Mesh] OR “Osteoporosis” [Mesh] OR osteoporosis [tiab] OR “Bone Density” [Mesh] OR “Bone Demineralization, Pathologic” [Mesh] OR “Bone Resorption” [Mesh] OR “Bone Development” [Mesh]) OR “Bone Remodelling” [Mesh] OR osteolysis [tiab] OR decalcification [tiab] OR bone [tiab] OR bones [tiab]).

MEDLINE® contains references of articles published since 1966, the majority of which are published in the USA. EMBASE also contains articles published in Europe, with references dating back to 1976. The Cochrane library contains over 250,000 records of Cochrane Controlled Trials [24].

#### Screening literature

Retrieved titles and abstracts were screened for inclusion eligibility and applicability by one researcher for search 1 and two separate researchers for search 2. Articles not related to the research question or not meeting inclusion criteria were discarded and the reason for exclusion was noted. Remaining articles were screened as full text and included in the final analysis if they met inclusion criteria. Abstracts concerning conference meetings were included in the search to prevent publication bias; however abstracts not containing adequate information related to research questions were discarded. Bibliographies of all included articles and of review articles that were excluded from the meta-analysis were reviewed for missed relevant articles.

#### Data extraction

Two investigators extracted data from all included articles for search 1 (author KEC) and for search 2 (author SD) using validated abstraction forms [Genetic Metabolic Dietitian International (GMDI)/Southeast Regional Collaborative (SERC) Evidence Abstract Worksheet (search 1) [25], Cochrane Renal Group protocol guidelines appendix 4 (search 2) [26]]. Data extracted included characteristics of study populations and control groups, study design, outcome measures, results, and limitations. Outcome measures of bone status were BMD [total body (TBMD), lumbar spine (LBMD) and/or femoral bone (FBMD)]; BTM; BMC; incidence or prevalence of osteopenia, osteoporosis, low BMD, or fractures; vitamin D and/or parathyroid hormone (PTH) status; and other indicators. DXA is the preferred and most commonly reported method to measure BMD in both children and adults [27-29]. Studies of bone in patients with PKU primarily report DXA estimates of BMD; however, other techniques such as pQCT, QUS and several X-ray methods used to measure BMD are available and are compared elsewhere [30,31]. We included studies measuring BMD using any recognized method.

#### Quality appraisal search 1

The Academy of Nutrition and Dietetics Evidence Analysis Process (AND EA Process [32]) was adapted by the GMDI/SERC effort to create nutrition management guidelines [25] for inborn disorders of metabolism and applied as the foundation for search 1. The AND Evidence Analysis Process provides a method to abstract data and assign a quality grade to primary and review articles retrieved through systematic searches. All included articles were reviewed, graded, and abstracted by author KEC, trained in the Evidence Analysis Process through participation in the development of PKU guidelines [33].

Quality criteria checklists (QCCs) were completed for all studies included in search 1. Each QCC included four relevance questions addressing the purpose and applicability of the study and 10 validity questions with a varying number of sub-questions (Tables 1 and 2). Answers to validity questions were used to assign a quality score of positive, negative or neutral to each article. For a positive quality rating, specific validity questions including an unbiased selection of patients, comparable study groups (i.e. matched controls for age, height and weight), sufficient description of study intervention and procedures, and clearly defined outcomes were required. Articles that did not meet these validity criteria, but did include other strengths were assigned a neutral quality rating. Articles that did not contain most of the validity components (6 out of 10 or more) received a negative quality rating.

**Table 1:** **Quality criteria checklist used in search 1— Primary research**

	**Relevance questions**
1.	Would implementing the studied intervention procedures (if found successful) result in improved outcomes for the patients/clients/population group? (N/A for some Epidemiological studies)
2.	Did the authors study an outcome (dependent variable) or topic that the patients/clients/population group would care about?
3.	Is the focus of the intervention or procedure (independent variable) or topic of study a common issue of concern to dietetics practice?
4.	Is the intervention or procedure feasible? (N/A for some Epidemiological studies)
	Validity questions
1.	Was the research question clearly stated?
1.1	Was the specific intervention(s) or procedure (independent variable(s)) identified?
1.2	Was the outcome(s) (dependent variable(s)) clearly indicated?
1.3	Were the target population and setting specified?
2.	Was the selection of study subjects/patients free from bias?
2.1	Were inclusion/exclusion criteria specified (e.g. risk, point in disease progression, diagnostic or prognosis criteria), and with sufficient detail and without omitting criteria critical to the study?
2.2	Were criteria applied equally to all study groups and/or all subjects?
2.3	Were health, demographics, and other characteristics of subjects described?
2.4	Were the subjects/patients a representative sample of the relevant population?
3	Were study groups comparable?
3.1	Was the method of assigning subjects/patients to groups described and unbiased? (Method of randomization identified if Randomized Controlled Trial (RCT))
3.2	Was the distribution of disease status, prognostic factors, and other factors (e.g. demographics) at baseline similar across study groups (original or created post hoc)?
3.3	Were concurrent controls used? (Concurrent preferred over historical controls.)
3.4	If cohort study or cross-sectional study, were groups comparable on important confounding factors and/or were preexisting differences accounted for by using appropriate adjustments in statistical analysis? (Criterion may not be applicable in some cross-sectional studies.)
3.5	If case control study, were potential confounding factors comparable for cases and controls? (If case series or trial with subjects serving as own control, this criterion is not applicable.)
3.6	If diagnostic test, was there an independent blind comparison with an appropriate reference standard (e.g., “gold standard”)?
4.	Was method of handling withdrawals described?
4.1	Were follow-up methods described and the same for all groups and/or all subjects?
4.2	Was the number, characteristics of withdrawals (i.e., dropouts, lost to follow up, attrition rate), and/or response rate (cross-sectional studies) described for each group? (Follow up goal for a strong study is 80%)
4.3	Were all enrolled subjects/patients (in the original sample) accounted for?
4.4	Were reasons for withdrawals similar across groups?
4.5	If diagnostic test, was decision to perform reference test not dependent on results of test under study?
5.	Was blinding used to prevent introduction of bias?
5.1	In intervention study, were subjects, clinicians/practitioners, and investigators blinded to treatment group, as appropriate?
5.2	Were data collectors blinded for outcomes assessment? (If outcome is measured using an objective test, such as a lab value, this criterion is assumed to be met.)
5.3	In cohort study or cross-sectional study, were measurements of outcomes and risk factors blinded?
5.4	In case control study, was case definition explicit and case ascertainment not influenced by exposure status?
5.5	In diagnostic study, were test results blinded to patient history and other test results?
6.	Were intervention/therapeutic regimens/exposure factor or procedure and any comparison(s) described in detail? Were intervening factors described?
6.1	In RCT or other intervention trial, were protocols described for all regiments studied?
6.2	In observational study, were interventions, study settings, and clinicians/provider described?
6.3	Was the intensity and duration of the intervention or exposure factor sufficient to produce a meaningful effect?
6.4	Was the amount of exposure and, if relevant, subject/patient compliance measured?
6.5	Were co-interventions (e.g., ancillary treatments, other therapies) described?
6.6	Were extra or unplanned treatments described?
6.7	Was the information for 6.4, 6.5, and 6.6 assessed the same way for all groups?
6.8	In diagnostic study, were details of test administration and replication sufficient?
7.	Were outcomes clearly defined and the measurements valid and reliable?
7.1	Were primary and secondary endpoints described and relevant to the question?
7.2	Were nutrition measures appropriate to question and outcomes of concern?
7.3	Was the period of follow-up long enough for important outcome(s) to occur?
7.4	Were the observations and measurements based on standard, valid, and reliable data collection instruments/tests/procedures?
7.5	Was the measurement of effect at an appropriate level of precision?
7.6	Were other factors accounted for (measured) that could affect outcomes?
7.7	Were the measurements conducted consistently across groups?
8.0	Was the statistical analysis appropriate for the study design and type of outcome indicators?
8.1	Were statistical analyses adequately described the results reported appropriately?
8.2	Were correct statistical tests used and assumptions of test not violated?
8.3	Were statistics reported with levels of significance and/or confidence intervals?
8.4	Was “intent to treat” analysis of outcomes done (and as appropriate, was there an analysis of outcomes for those maximally exposed or a dose–response analysis)?
8.5	Were adequate adjustments made for effects of confounding factors that might have affected the outcomes (e.g., multivariate analyses)?
8.6	Was clinical significance as well as statistical significance reported?
8.7	If negative findings, was a power calculation reported to address type 2 error?
9.	Are conclusions supported by results with biases and limitations taken into consideration?
9.1	Is there a discussion of findings?
9.2	Are biases and study limitations identified and discussed?
10.	Is bias due to study’s funding or sponsorship unlikely?
10.1	Were sources of funding and investigators’ affiliations described?
10.2	Was there no apparent conflict of interest?

**Table 2:** **Quality criteria checklist used in search 1 — Reviews**

	**Relevance questions**
1.	Will the findings of the review, if true, have a direct bearing on the health of patients?
2.	Is the outcome or topic something that patients/clients/population groups would care about?
3.	Is the problem addressed in the review one that is relevant to dietetics practice?
4.	Will the information, if true, require a change in practice?
	Validity questions
1.	Was the research question clearly focused and appropriate?
2.	Was the search strategy used to locate relevant studies comprehensive? Were the databases searched and the search terms use described?
3.	Were explicit methods used to select studies to include in the review? Were inclusion/exclusion criteria specified and appropriate? Were selection methods unbiased?
4.	Was there an appraisal of the quality and validity of studies included in the review?
5.	Were specific treatments/interventions/exposures described? Were treatments similar enough to be combined?
6.	Was the outcome of interest clearly indicated? Were other potential harms and benefits considered?
7.	Were processes for data abstraction, synthesis, and analysis described? Were they applied consistently across studies and groups? Was there appropriate use of qualitative and/or quantitative synthesis? Was variation in findings among studies analyzed? Were heterogeneity issues considered? If data from studies were aggregated for meta-analysis, was the procedure described?
8.	Are the results clearly presented in narrative and/or quantitative terms? If summary statistics are used, are levels of significance and/or confidence intervals included?
9.	Are conclusions supported by results with biases and limitations taken into consideration? Are limitations of the review identified and discussed?
10.	Was bias due to the review’s funding or sponsorship unlikely?

#### Assessment of bias search 1

QCC-derived quality ratings reflected the likelihood of bias in each study. Those rated positive were unlikely to contain significant bias, while those with neutral quality ratings included some elements likely to produce bias. Negative quality ratings indicated that bias in the study was very likely and these articles were excluded from the review.

#### Quality appraisal search 2

Quality appraisal and assessment of bias were performed for search 2 on all assessed full text articles by two separate researchers (SD and AMB) and outcomes were discussed. The ‘Scottish Intercollegiate Guidelines Network’ (SIGN) checklists were used [34] to assess quality based on the study design (RCT, cohort or case–control study). SIGN checklists are based on the Grading of Recommendations Assessment, Development and Evaluation (GRADE) [35] approach. Articles were appraised as of low, acceptable or high quality and those assessed as low quality were excluded from the review.

#### Assessment of bias search 2

Quality ratings reflected the likelihood of bias in each study. Those rated high quality were unlikely to contain significant bias, while those with acceptable quality ratings included or did not include some elements likely to produce bias. Low quality ratings indicated bias in the study was likely and these studies were not included in the review.

#### Statistical analysis

Z-scores and T-scores for BMD are calculated to clinically assess an individual’s bone status. T-scores describe the number of standard deviations (SD) by which a patient’s BMD differs from the expected mean value in a healthy young adult. The World Health Organization (WHO) defines osteopenia in adults as a T-score between -1 and -2.5, and osteoporosis as a T-score below -2.5 [29]. Z-scores describe the number of SDs by which the BMD in an individual differs from the mean value expected for age and sex. For children, Z-scores are mostly used. The International Society for Clinical Densitometry (ISCD) states that the diagnosis of osteoporosis in children, premenopausal women and males under 50 years of age should not be based on densitometric criteria alone. Instead, a BMD Z-score below -2 is defined as “BMD below the expected range for age” or “low BMD for chronological age” and cannot be defined as osteoporosis unless coupled with a significant fracture history [28]. The ISCD does however stress that Z-scores above -2 do not preclude the possibility of skeletal fragility. Most recent studies of bone health in PKU report BMD Z-scores only [14,36], because the patient population is relatively young and pediatric patients are assessed together with adult patients. BMD can be measured at a variety of locations and we included studies that measured BMD in total body, spine and femur. Spinal BMD reflects BMD in trabecular bone, and femoral BMD is significant for cortical bone [37].

Qualitative and quantitative analyses were performed to assess BMD in patients with PKU. Qualitative analysis was performed to review evidence on bone health and assess the prevalence of low BMD in patients with PKU. Quantitative analysis was performed in the form of a meta-analyses to analyse whether if BMD Z-scores are different in patients with PKU than reference values (deviant from 0 SD of reference). If the full text of an article did not contain BMD or BMD Z-scores, the authors were contacted to obtain data. The meta-analysis was performed in Review Manager [38]; a fixed effects model or random effects model was used to pool the patient-based data. The choice for selecting a fixed effects model or a random effects model was based on heterogeneity of the data per meta-analysis. Low heterogeneity between studies led to the use of a fixed effects model, and high heterogeneity to the use of a random effects model to pool data [39,40]. Heterogeneity was tested by calculating I^2^ (heterogeneity is low when I^2^ is ≤ 25%, moderate if 25 ≤ 50% and high if ≥ 75%) [39]. The presence of publication bias was visually assessed by means of a funnel plot and calculation of Egger’s test with statistical software (IBM SPSS statistics 20) [40]. By using outcomes from a specific population (early diagnosed and treated patients with PKU), it should be noted that the effect cannot be extrapolated to other patients with PKU.

To assess BMD Z-scores considered low for chronological age (below -2), we used a normal distribution curve to estimate prevalence in a healthy population and developed a PKU-specific normally distributed curve based on estimated effect size for LBMD. Since these estimates are hypothetical, we also calculated prevalence in a normal population using the 2007–2008 National Health and Nutrition Examination Survey (NHANES) [41] data. We limited the analysis to participants 8–45 years of age with lumbar spine and femoral neck BMD measurements since NHANES does not collect data in children under eight years of age, and included studies in this review do not report on patients over the age of 45 years (older patients are often late diagnosed). Z-scores for age and sex were calculated using the Centers for Disease Control and Prevention’s (CDC) references [42] for lumbar spine and femoral neck BMD. We used the computer program SAS-Callable SUDAAN 11.0.1 to calculate weighted population prevalence of low BMD for chronological age (Z-score <-2), taking into account primary sampling units and strata. To calculate final prevalence, we limited analyses to a subpopulation of non-Hispanic Caucasian participants only.

All other outcomes including BTM, BMC, and blood vitamin D and PTH status were evaluated qualitatively. Overview tables of results were generated to summarize results and draw conclusions. Factors examined in association with bone-related outcomes were included in overview tables with statistical methods, direction of association, and statistical and clinical significance. If analyses controlled for variables, these were also noted. Factors significantly associated with bone-related outcomes in multiple studies were noted to identify potential underlying causes of low BMD. Finally, all data collected and summarized through quality appraisal and overview tables were used to identify gaps in the bone health evidence base in PKU.

## Results

### Study selection

**Figure 1:**
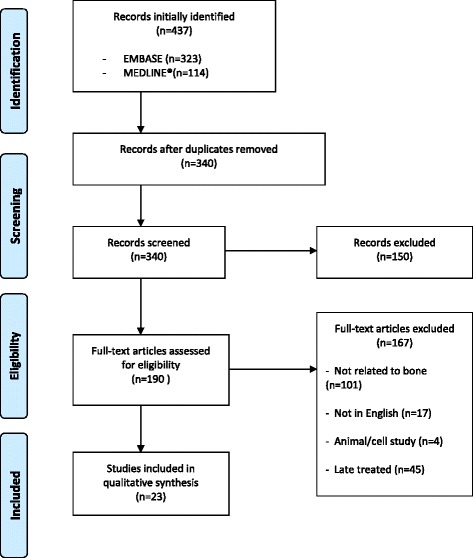
**Inclusion process flow diagram for search 1 (PRISMA 2009** **[** 22 **]** **).**

Twenty-three articles were included in this review as a result of study selection criteria for search 1, resulting from 437 initially identified records from both EMBASE and MEDLINE® (Figure 1).

**Figure 2:**
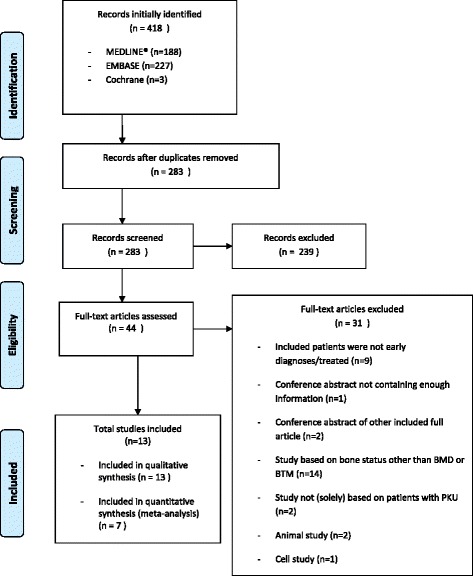
**Inclusion process flow diagram for search 2 (PRISMA 2009** **[** 22 **]** **).**

A total of 13 articles were included for search 2 after selection from 418 initially identified records from EMBASE, The Cochrane Library and MEDLINE® (Figure 2).

### Included studies

Articles included in search 1 and search 2 were combined and 23 unique articles were identified. We found that both study teams included 13 records (57%), listed in Additional file 1: Table S1 with their study characteristics. Studies were published between 1994 and 2013, with the majority published since 2000 (69%). Twelve were cohort studies whereas one was a case–control study. Search 1 included 10 articles that were not included in search 2. Most of these articles were published before 2000 (80%) and identified due to differences in search terms (n = 5) or discrepancy in quality ratings (n = 5). Of these 10 articles, 5 were discarded when appraised as low quality in search 2 and the remaining 5 articles did not address BMD and were therefore excluded in search 2. We limited our analyses to articles included in both searches only (n = 13).

### Quality appraisal

**Figure 3:**
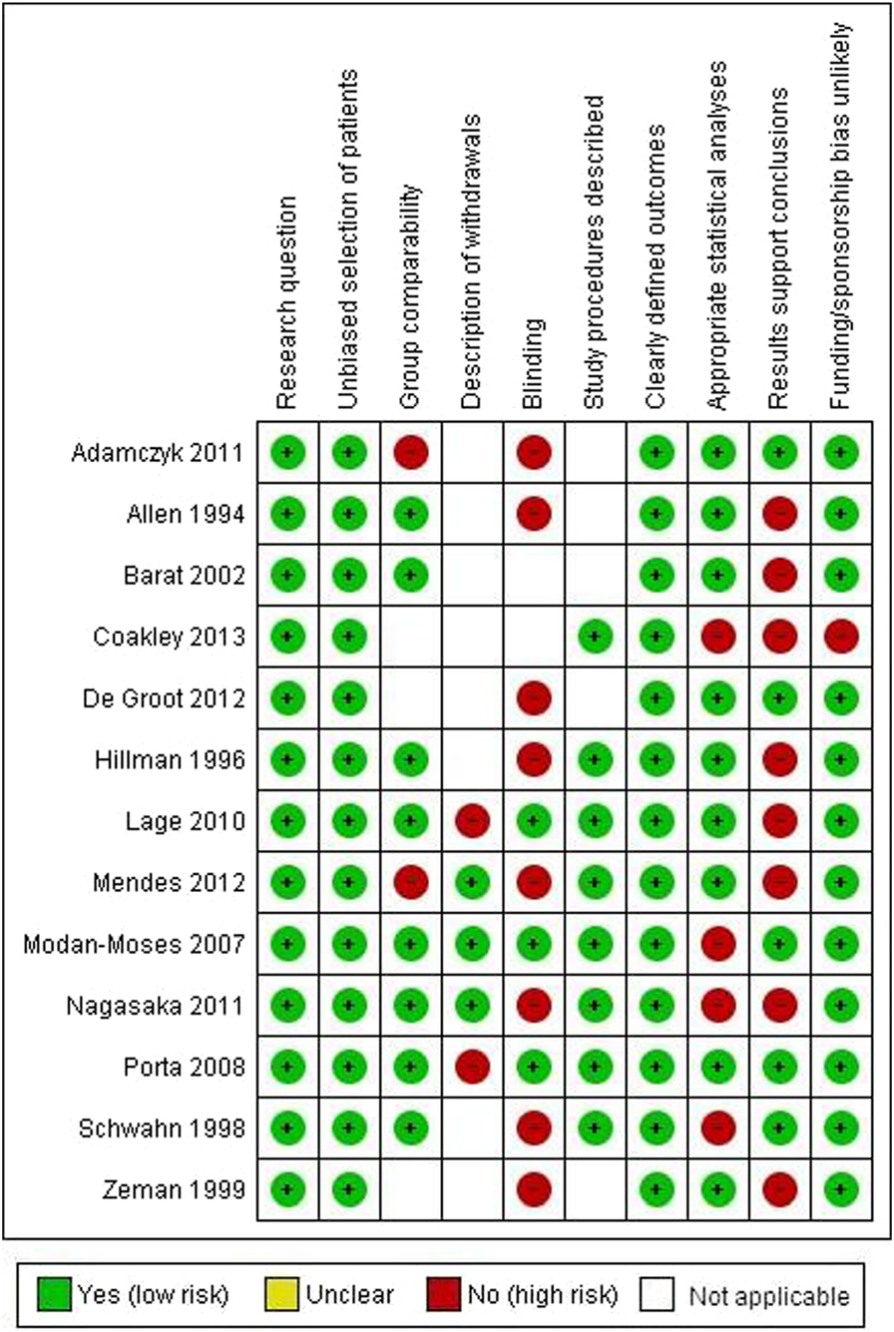
**Risk of bias summary table search 1.**

**Figure 4:**
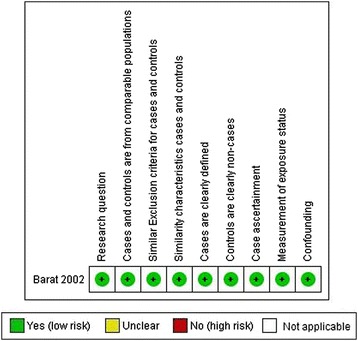
**Risk of bias summary table case–control study search 2.**

**Figure 5:**
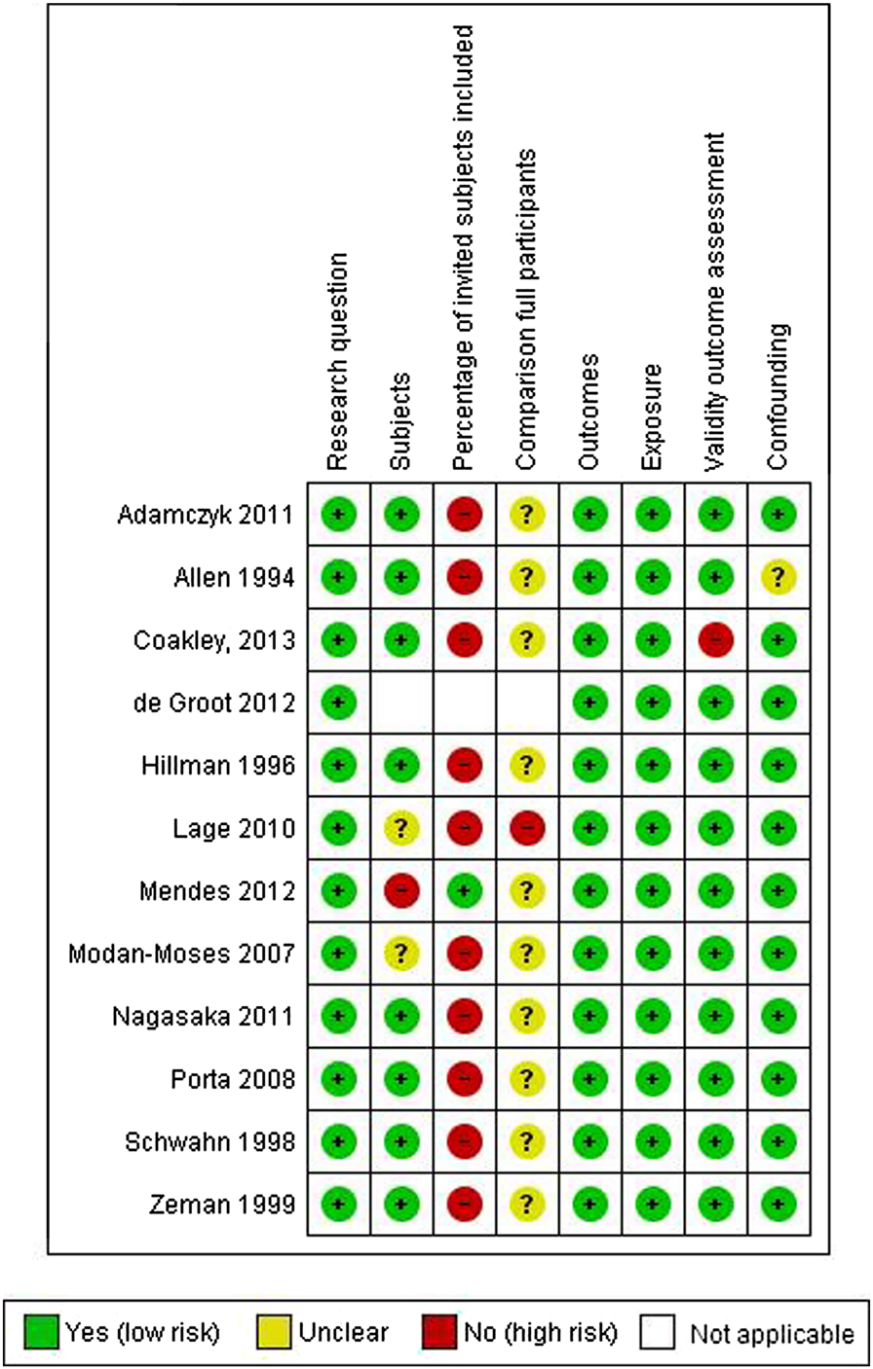
**Risk of bias summary table cohort studies search 2.**

Of the 13 included studies, seven (54%) were graded neutral quality and six (46%) were graded positive quality according to the AND Evidence Analysis Process applied during search 1 (Figure 3). As defined by SIGN checklists, applied during search 2, the majority of papers were graded acceptable quality (n = 11) and two [12,21] were high quality (Figures 4 and 5).

While quality was determined on separate scales by each search team, scores of eight articles (62%) corresponded between AND and SIGN quality scores. Seven of the eight were assessed as neutral quality by AND criteria in search 1 and a corresponding acceptable quality by SIGN checklists in search 2. One article was graded positive quality by AND criteria and a corresponding high quality by SIGN checklist. The remaining five of the 13 (38%) included studies with quality ratings that did not agree between AND and SIGN scales. All five were scored with the highest quality rating (positive) by AND criteria in search 1, but rated acceptable quality by SIGN checklists in search 2. Most of these papers did not fully describe the number of patients recruited for the study versus the number of actual study, a requirement for a high quality rating on the SIGN checklist. Two articles that were scored positive based on AND criteria also scored very well on the SIGN checklists, but they were retrospective studies, automatically disqualifying them for a rating above acceptable quality [14,43].

### BMD in early treated patients with PKU

A total of 11 articles studied BMD in early treated patients with PKU; 10 cohort studies and one case–control study. Combined, a total of 360 patients (range 11 – 57 per study) were included. Five studies included pediatric patients only, one study selectively included adult patients [44], and 5 studies included pediatric and adult patients. Ten of the 11 studies found that BMD was significantly lower in patients with PKU compared to a reference group or controls. A single study, including children and adolescents, did not find altered BMD in patients with PKU [20].

#### BMD in pediatric patients with PKU

All 5 studies that included pediatric patients used DXA to measure BMD. Four reported a reduced BMD in patients and one study did not find a significantly altered LBMD when comparing 8 pediatric patients with PKU to a control population [20]. In this study, however, two of the eight (25%) patients had a LBMD Z-score below -2, meeting the criteria for low BMD for pediatric patients defined by the ISCD [28,45]. Both are described as adolescent patients not adherent to diet.

Of the 4 articles reporting lower BMD in patients with PKU, Adamczyk et al. [13] described a group of 45 children (mean age 13.8 ± 5.2 years) and concluded that skeletal status is impaired in patients with PKU (mean Z-score LBMD -0.572 ± 1.270 and TBMD -0.117 ± 1.347). They also found that in patients who were sexually mature, those who were non-adherent to diet had a significantly lower BMD than those who adhered to diet.

Furthermore, Barat et al. [43] investigated a group of 13 pediatric patients with PKU, reporting a mean LBMD Z-score of -1.36 ± 1.586.

Similarly, a study by Hillman et al. [37] established that BMD at multiple sites was significantly lower in a group of 11 pediatric patients with PKU compared to age-matched controls [LBMD 0.61 ± 1.5 g/cm^2^ vs 0.72 ± 0.24 g/cm^2^ and FBMD 1.56 ± 0.30 g/cm^2^ vs 1.87 ± 0.56 g/cm^2^].

Finally, Allen et al. [17] investigated 32 pre-pubertal patients (mean age 7.7 ± 2.3 years) and found significantly lower BMD compared to age-matched, non-PKU controls (TBMD 0.770 ± 0.085 g/cm^2^ vs 0.814 ± 0.075 g/cm^2^ and LBMD 0.619 ± 0.100 g/cm^2^ vs 0.701 ± 0.097 g/cm^2^). TBMD of patients with PKU was 97.1% of predicted BMD for children of the same gender and age while LBMD was 92% of predicted BMD. Clinical fracture risk was not directly evaluated by any of the studies

#### BMD in pediatric and adult patients with PKU

Four of the 5 studies that described a mixed group of pediatric and adult patients used DXA to assess BMD and 1 study [12] used pQCT to assess BMD in the radius. All 5 studies reported altered BMD in patients with PKU.

A conference abstract by Coakley et al. [46] reported TBMD in a population of 57 patients over 4 years of age. The authors found that 16 patients (28%) had a TBMD Z-score between -1 and -2.5 and three patients (5%) had a Z-score below -2.5. TBMD was positively correlated with age (controlling for BMI, sex, metabolic control, and medical food intake) in their population with a mean age of 17.5 years. Similar results were obtained by three other studies. de Groot et al. [14] reported a mean LBMD Z-score of -0.78 ± 1.1 in a group of 53 patients with PKU and low BMD (LBMD Z-score below -2) in 10 patients (19%). A subgroup analysis showed that younger patients had a higher prevalence of low BMD though no significant correlations were established between BMD and age.

Lage et al. [15] investigated BMD in 47 patients with PKU and found a mean Z-score significantly below 0 (mean FBMD Z-score -1.2 ± 1.0; LBMD Z-score -0.4 ± 0.8). A Z-score between -1 and -2.5 was found in 13 patients (28%) and a Z-score below -2.5 in 6 patients (13%) of at least one site. The authors found a negative correlation between age and LBMD in patients 6–10 years of age and a positive correlation between age and FBMD in patients 11–18 years of age.

Zeman et al. [16] studied 44 patients with PKU and described that 14 (32%) had a TBMD Z-score below -1 and 20 patients (45%) had a LBMD Z-score below -1, of whom 6 had a Z-score below -2.5. No correlation between age and LBMD or TBMD was evident.

A final study by Schwahn et al. [12] used pQCT in 14 patients with PKU ages 5–28 years to assess BMD of both spongy and total bone of the non-dominant distal radius. They found that spongy bone BMD was significantly lower in patients with PKU compared to 14 age, gender, weight and height-matched controls [139.7 ± 23.5 mg/cm^3^ vs 169.3 ± 31.5 mg/cm^3^]. Mean total bone BMD of the radius in patients with PKU was slightly lower than controls, but not significant. Within the group of PKU patients, TBMD and LBMD were lower in adolescents ages 13–16 years compared to younger children and adults. The authors hypothesized that patients with PKU have altered trabecular bone architecture indicated by low spongy bone BMD and/or altered mineralization, but show minor changes of cortical bone. They emphasize this hypothesis by describing the case of an untreated severely retarded female patient who showed lower BMD, especially of trabecular bone, at 10 years of age, which could not be explained by a history of malnutrition or immobilization.

#### BMD in adult patients with PKU

The only study included in this review examining exclusively adult patients is by Modan-Moses et al. [44]. In a group of 31 patients, 42% had compromised BMD (Z-score <-1). Mean TBMD Z-scores (-0.474 ± 0.719) and FBMD Z-scores (-0.727 ± 0.66) were significantly lower than expected for individuals of the same sex and age without PKU (p = 0.002 and p < 0.001, respectively). Mean LBMD was also lower than expected, but not statistically significant.

#### Prevalence of compromised bone status

Five studies examined the prevalence of low BMD. In cohort studies, prevalence of osteopenia (defined in all papers as a Z-score between -1 and -2.5) ranged from 28–46% [15,16,20,44,46]. A single study estimated the prevalence of osteopenia retrospectively, finding 62% of children with PKU had a Z-score between -1 and -2.5 at age 12 [43]. The prevalence of osteoporosis (defined in each study as a BMD Z-score below -2.5) ranged from 5–14% [15,16,20,44,46]. A single study defined low BMD as a Z-score below -2, consistent with ISCD recommendations, and reported a prevalence of 19% in children and adults [14]. Seven studies included in this review did not report the prevalence of low BMD [12,13,17,19-21,37], and none of the 13 studies reported BMD T-scores.

In known literature databases, we found no reports on low BMD prevalence (Z-score <-2) in a reference population of adolescents or young adults for comparison. A self-performed pilot analysis of weighted NHANES data [41], however, suggests Z-scores between -1 and -2.5 are found in 14.9% (95% CI 12.6–17.4%) at the proximal femur and 14.3% (95% CI 12.1–17.0%) at the lumbar spine in non-Hispanic Caucasians ages 8–45 years. Z-scores below -2.5 were found in an additional 0.13% (95% CI 0.03–0.53%) at the proximal femur and 0.53% (95% CI 0.13–2.10%) at the lumbar spine. The prevalence of low BMD for chronological age, defined by ISCD criteria as a Z-score below -2, was 1.8% (95% CI 1.0–3.3%) at the lumbar spine and 1.6% (0.8–3.0%) at the proximal femur in NHANES data. These findings confirm a normal distribution of BMD in the general population, in which a 2.3% of the population would be expected to have a score below -2 SD.

#### Height-corrected bone mineral density

Three studies included a height-correction of DXA measured BMD to correct for height bias.

Adamczyk et al. [13] reported SD-scores on DXA results with correction for patient height and gender. TBMD and LBMD SD scores were significantly lower in adolescent patients who were not compliant with diet compared to compliant patients. Total body and lumbar spine BMC SD scores were also significantly lower in non-compliant versus compliant patients.

Allen et al. [17] reported lower LBMD in children with PKU compared to controls, adjusting for height and weight. There was no difference in mean age and SD height and weight scores between the PKU and control children. Based on predictions for LBMD derived from control data, LBMD of the children with PKU was 92% of what was expected.

De Groot et al. [14] report a positive correlation between BMD and height in children with PKU under age 18, but not in adults. They conclude Z-scores of BMD found in their whole study population (n = 53; mean age 16.7 ± 9.1) are not significantly correlated to height and weight.

#### Blood Phe levels and BMD

Nine studies investigated the correlation between Phe blood levels and BMD [3,13-17,37,44,46], seven of which found no correlation [14-17,37,44].

de Groot et al. [14] found no significant correlations between BMD and frequency or proportion of Phe blood concentrations below the recommended threshold, or the mean cumulative variation of blood Phe concentrations.

Two studies, however, did find a negative correlation between Phe levels and BMD [13,43]. First, Barat et al. [43] describe that although mean Phe concentration did not correlate with BMD outcomes, patients with BMD Z-scores below -1 had a significantly higher mean cumulative Phe variation than controls [3.1 ± 0.4 mg/dl versus 2.5 ± 0.4 mg/dl (p-value = 0.006)]. Based on their findings, the researchers suggest variations of Phe concentrations may contribute to lower BMD in children with PKU. Second, Adamczyk et al. [13] report that among pediatric patients who had reached sexual maturity, those who were compliant to diet had significantly higher BMD Z-scores, and lower plasma Phe levels than non-compliant patients. A regression analysis also showed serum Phe concentration had the most negative influence on BMD values of all variables examined including demographics such as age, sex, and body mass index (BMI).

#### Dietary intake and BMD

Seven cohort studies examined the impact of total protein and/or medical food protein intake on BMD [13,15-17,37,44,46]. Evidence is consistent for total protein intake with 4 studies reporting no correlation with BMD [17,37,44,46]. Studies assessing medical food protein intake and BMD, however, are inconsistent. Coakley et al. [46] found a positive correlation between medical food prescription (grams of protein per day) and actual medical food intake and TBMD. Zeman et al. [16] reported no correlation between daily intake of Phe-free amino acid mixture per kilogram body weight and TBMD or LBMD Z-scores.

### Meta-analysis of BMD in early treated patients with PKU

A meta-analysis was performed on mean BMD Z-scores in the spine (7 studies), whole body (3 studies) and femur (2 studies). All studies used DXA to measure BMD. Pooling of data was performed by using available BMD Z-scores provided by the authors, either in the article [14,20,44,46] or through added information on request per e-mail [13,15,43,46]. A fixed or random effects model of generic inversed variance was used to examine the mean difference between patients with PKU and normal values for healthy age and sex-matched controls (BMD Z-score = 0).

#### Exploration of heterogeneity

**Figure 6:**
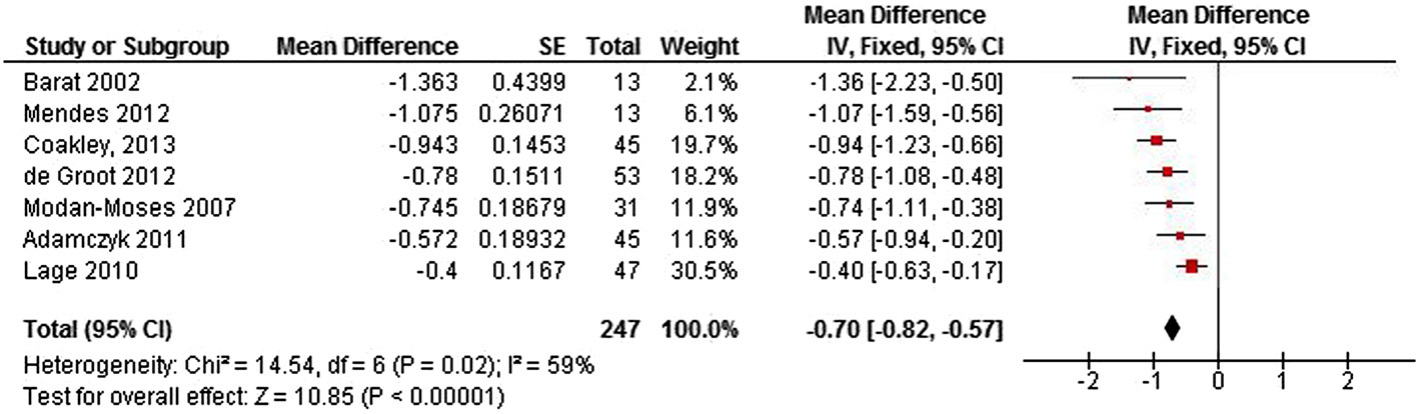
**Forest plot of LBMD (Z-score) in patients with Phenylketonuria.** (SE = standard error, IV = Inverse Variance, CI = confidence interval).

Seven papers measured LBMD for a total of 247 patients [13-15,20,43,44,46]. Mean Z-scores ranged from -1.363 to -0.4 (Figure 6). A moderate heterogeneity was observed (I^2^ = 59%), justifying the pooling of results and the use of a fixed effects model [39,47].

**Figure 7:**
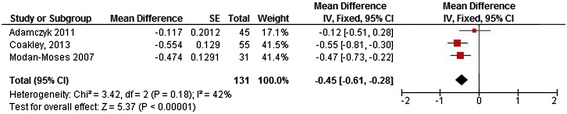
**Forest plot of TBMD (Z-score) in patients with Phenylketonuria.** (SE = standard error, IV = Inverse Variance, CI = confidence interval).

Three papers measured TBMD for a total of 133 patients [13,44,46]. Mean Z-scores ranged from -0.55 to -0.12 (Figure 7). A moderate heterogeneity was seen (I^2^ = 42%), justifying the pooling of results by the use of a fixed effects model [39,47].

**Figure 8:**
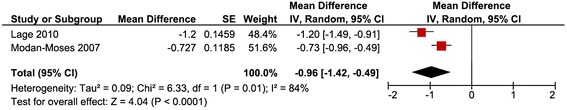
**Forest plot of FBMD (Z-score) in patients with Phenylketonuria.** (SE = standard error, IV = Inverse Variance, CI = confidence interval).

Two papers were available providing FBMD for a total of 78 patients [15,44]. Mean Z-scores ranged from-1.2 to-0.727 (Figure 8). High heterogeneity between the two studies was observed (I^2^ = 84%), probably due to the low amount of included studies, therefore a random effects model was used to pool patients-based results [39,47].

#### Assessment of publication bias

**Figure 9:**
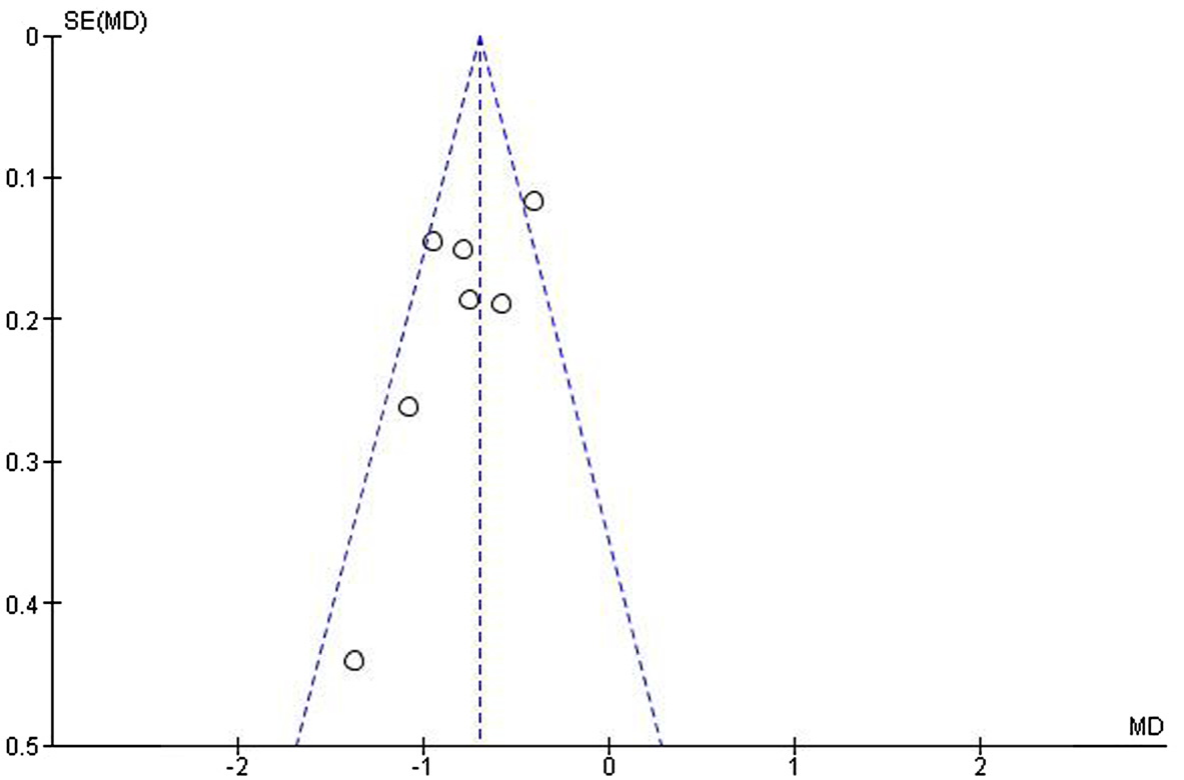
**Funnel plot LBMD (Z-score) in patients with PKU.** (SE = standard error; MD = mean difference) (Egger’s test: p = 0.407).

There was no evidence of publication bias for LBMD as visual assessment of the funnel plot (Figure 9) shows a symmetrically distributed inversed funnel and Egger’s test was not significant (p = 0.407). Evaluation of publication bias by funnel plot or Egger’s test for TBMD and FBMD was not reliable due to the limited number of studies included.

#### Pooled patient-based BMD

In 247 pooled patients with PKU included in 7 studies, mean LBMD Z-score was -0.70 (95% CI-0.82,-0.57). The overall effect is significantly different (P < 0.00001) from the norm and none of the individual studies crossed the line of no effect (Figure 6).

In 133 pooled patients with PKU included in 3 studies, mean TBMD Z-score was -0.45 (95% CI-0.61,-0.28). The overall effect is significantly (P < 0.00001) different from the norm (Figure 7). One of the individual studies crossed the line of no effect [13].

In 78 pooled patients with PKU included in 2 articles, mean FBMD Z-score was -0.96 (95% CI-1.42,-0.49). The overall effect is significantly different (P < 0.0001) from the norm and both included studies do not cross the line of no effect (Figure 8).

### Bone turnover markers in early treated patients with PKU

Four cohort studies examined BTM in patients with PKU. Combined, a total of 110 patients (range 11 – 45 per study) were included. Two studies included only pediatric patients [13,37], one study included only adult patients [21] and one study included both pediatric and adult patients [48]. All included studies found significant alterations in one or more BTM.

Adamczyk et al. [13] measured 3 bone formation markers including carboxyterminal telopeptide of type I collagen (P1NP), bone-specific alkaline phosphatase (bALP) and osteocalcin in serum of 45 pediatric patients with PKU. They compared BTM by subgroups of patients based on sexual maturity and compliance to diet, but did not provide mean values for the group as a whole. Among those compliant with diet, sexually immature patients (Tanner stage below 5) had higher P1NP (10.33 ± 2.97 lg/l vs 6.62 ± 2.10 lg/l) and bALP (75.67 ± 49.60 U/l vs 30.67 ± 37.05 U/l) compared to sexually mature patients.

On the other hand, among sexually mature patients, differences were found between non-compliant and compliant patients including higher bALP (63.0 ± 46.43 U/l vs 30.67 ± 37.05 U/l) and higher osteocalcin (48.87 ± 23.0 ng/ml vs 33.15 ± 11.88 ng/ml). These findings are in line with physiological concentrations of BTM, which are increased during active periods of bone remodeling including growth in childhood and pre-pubertal adolescence [49,50].

Hillman et al. [37] assessed BTM in 11 children with PKU in comparison to 11 age-matched controls. Bone formation markers bALP (6.1 ± 6.3 U/l vs 13.1 ± 2.0 U/l) and osteocalcin (72 ± 30 U/l vs 126 ± 43 U/l) were significantly lower in patients with PKU compared to controls, whereas P1NP was lower (290 ± 174 U/l vs 400 ± 159 U/l) but not significant. Bone resorption markers including urinary tartrate resistant acid phosphatase and calcium creatinine ratio did not differ between subjects and controls.

Nagasaka et al. [21] reported BTM in adult patients (n = 34) compared to age-matched controls (n = 36). The bone resorption markers blood pyridinoline cross-linked telopeptide domain of type I collagen, urinary deoxypyridinoline, and urinary N-telopeptide of type I collagen were significantly higher in patients with PKU than in the control group. Blood osteoprotegerin, an inhibitor of bone resorption, was also significantly lower in individuals with PKU. No differences were found in the bone formation markers bALP and osteocalcin between the PKU and control groups.

Porta et al. [48] examined spontaneous osteoclastogenesis, the differentiation of mature osteoclasts from precursors to initiate the process of bone resorption, in pediatric and adult patients with PKU compared to 20 age and sex-matched controls. Their results show that osteoclasts, generated through spontaneous osteoclastogenesis from peripheral blood monocytes, were larger and nearly double in number compared to those of control subjects.

#### Blood Phe levels and BTM

Four studies investigated correlations between blood Phe concentrations and individual BTM [13,21,37,48]. Bone formation markers including bALP and osteocalcin were reported as higher in patients with Phe above recommended levels compared to patients with recommended Phe levels [13]. Moreover, mean serum Phe over a period of one year was significantly correlated with the number of osteoclasts, indicators of active bone resorption, in patients with PKU (r = 0.576; p-value = 0.010) [48]. Other studies report no correlation between serum Phe concentrations and BTM [21,37].

### Other indicators of bone status in early treated patients with PKU

#### Bone mineral content

BMC was examined in a single study [13] by Adamczyk et al. (2011). The authors reported higher total body BMC and spine BMC in mature patients with concurrent recommended threshold Phe levels at time of measurement compared to mature patients with Phe levels above recommendations. Moreover, in non-compliant patients, the total body BMC to lean body mass (LBM) ratio was reduced, an indicator of increased risk for fragility fractures. In compliant patients, however, the BMC/LBM ratio was not different than expected for age and height.

#### Vitamin D status in patients with PKU

Six included studies measured blood vitamin D status, all are cohort studies [14,15,21,37,44,46]. Among the cohort studies, findings varied by age group. One study of 31 adults with PKU showed that all patients had normal 25-hydroxyvitamin D concentrations, the primary indicator of vitamin D status [44]. Two studies report associations between vitamin D and indicator of bone status in children and adults with PKU [14,46], but do not mention the prevalence of vitamin D deficiency or insufficiency. A case–control study suggests 25-hydroxyvitamin-D and 1,25-dihydroxyvitamin-D concentrations in children with PKU do not differ from controls matched on sex and age [37]. In male and female adults with PKU on the other hand, 1,25-dihydroxyvitamin-D was reported as significantly higher than in controls and 25-hydroxyvitamin-D was significantly lower than controls [21]. Coakley ea. report a significant positive association between TBMD and 1,25-dihyroxyvitamin D [46]. All other studies report no correlation between plasma 25-hydroxyvitamin-D and BMD at any site [14,15,44].

#### Parathyroid Hormone (PTH) in patients with PKU

Four of the 13 included studies measured PTH, all are cohort studies [13,21,37,44]. Overall, children with PKU have similar PTH concentrations to healthy controls [37], but differences are reported in subgroups. PTH appears to be significantly higher in non-compliant children and adolescents compared to those with recommended Phe levels [13]. PTH is also reported to be higher in female and male adults with PKU compared to controls, but the difference is not statistically significant in males [21]. PTH above the normal reference range was reported in two of 31 (6%) adults with PKU examined in one study [21].

#### Other indicators of bone status

Fracture history was examined in a single study. Modan-Moses et al. [44] reported that 4 patients (13%) included in their study had a significant fracture history, though all were the result of physical trauma. Two patients had normal BMD, one had a LBMD Z-score of -1.9, and one had a FBMD Z-score of -2.4. Greeves et al. [51] provided the first investigation of fractures in patients with PKU, reporting the risk of fracture is 2.6 times greater in patients with PKU over 8 years of age compared to controls. Though the study did not meet inclusion criteria for this review, Greeves ea. provides the only estimate of fracture risk in known literature on patients with PKU.

Concentrations of vitamins and minerals related to bone metabolism, including calcium, phosphorus and magnesium, were also measured in several studies.

Calcium was measured in six of the 13 included studies [13-15,21,37,44]. Serum calcium was reported as significantly lower in children with PKU compared to controls [37], although no difference in total calcium was found between compliant and non-compliant subgroups of children and adolescents with PKU [13]. In adults with PKU, urinary calcium excretion was significantly higher than in controls [21], though all patients’ blood calcium concentrations fell within normal range [44]. A negative correlation between blood calcium and BMD Z-score in individuals with PKU of all ages was reported by de Groot et al. [14], but no correlation between plasma calcium and BMD Z-score was found by Lage et al. [15].

Phosphorus was measured in four of the 13 included studies [14,21,37,44]. Children and adults with PKU were reported to have serum phosphorus concentrations within normal ranges and comparable to healthy reference groups [37,44]. While no difference in phosphorus concentration was found between adults with PKU and controls [21], children with PKU had lower phosphorus excretion and higher phosphorus reabsorption compared to controls [37]. Children and adults with low BMD Z-scores were described to have higher blood phosphorus concentrations compared to those with normal BMD, but the correlation between blood phosphorus and BMD Z-score was not significant [14].

Two studies examined serum magnesium and found lower concentrations in children with PKU compared to controls [14,37]. Children and adults with PKU with low BMD also had lower, though not significant, magnesium than those with normal BMD [14]. Magnesium did not correlate significantly with BMD Z-score [14] or any measure of bone status [37] in either study.

## Discussion

### BMD in early treated patients with PKU

The results of our qualitative and quantitative review suggest that mean BMD is lower in PKU patients compared to reference groups but within the normal range in most patients, thus the clinical relevance of this finding is questionable.

The meta-analysis of pooled data from 247 patients with PKU shows an overall effect size for LBMD Z-score of -0.70 (95% CI-0.82,-0.57). The overall effect size for TBMD Z-score in 133 patients is also below zero [-0.45 (95% CI-0.61,-0.28)]; however one of the studies [13] shows a large range and crosses the no effect line (Z-score = 0). Because heterogeneity is moderate, it can be assumed the overall effect size is reliable and that TBMD Z-score in patients with PKU is indeed below 0. Our meta-analysis for FBMD shows a similar effect, although heterogeneity of the populations and outcomes in these studies hamper a firm conclusion.

In our qualitative analysis of BMD Z-scores in patients with PKU, we found study-defined prevalence of osteopenia and osteoporosis [15,16,20,44,46] to be higher than prevalence of comparable Z-score categories in a reference population of adolescents and young adults; however, our meta-analysis of pooled BMD Z-scores reported in patients with PKU challenges this hypothesis. Overall effect sizes of Z-scores for LBMD, TBMD and FBMD calculated in our meta-analyses are categorized as normal by ISCD standards. The 95% confidence intervals of effect size for separate studies do, however, show a number of patients with LBMD Z-scores below -2. Thus, a subset of patients with PKU reported in articles included in this study might have a higher risk for skeletal fragility and fractures. Based on the assumptions that our data are normally distributed and the overall effect size for LBMD Z-score is -0.70, approximately 10% of early treated patients with PKU may have a LBMD below -2 SD. This means 90% of early treated patients with PKU are not at risk for low BMD, a much better outcome than expected from single studies from the literature. The projected 10% of patients with a Z-score below -2 may be at risk for osteoporosis and may benefit from the same preventative and treatment strategies defined for healthy individuals [52].

A large limitation to these findings is the lack of standardization between individual study’s definitions of osteopenia and osteoporosis and clinical diagnostic criteria. In pediatric patients, fracture history must be assessed alongside BMD Z-score before diagnosis can be made. In adult patients, WHO guidelines require T-scores to diagnose osteopenia or osteoporosis. Currently, most studies in patients with PKU report Z-scores, regardless of age groups studied, and only 2 studies report fracture history, but do not mention its relevance to clinical diagnoses. Thus, studies reporting prevalence of osteopenia and osteoporosis in patients with PKU are missing essential information necessary to qualify patients for these diagnoses.

We also examined the impact of Phe status and dietary compliance on BMD. Most studies researching correlations between Phe values and BMD did not find a correlation. Dietary compliance and dietary intake assessed as reported medical food intake [16,46], total protein [17,37,44] or Phe intake [16,17] were not correlated to BMD or BTM. The impact of overall protein status, including biological value of intact versus medical food protein and percent of total protein derived from medical food, on bone were not considered by any studies.

Age in relation to BMD was examined, but outcomes are very heterogeneous with associations varying across age groups. We are not able to draw conclusions about BMD in different age categories based on the included studies; however children under the age of 10 years and those from 13–16 years of age may have a higher prevalence of low BMD than other age groups [12,14].

### Bone turnover in early treated patients with PKU

Results on bone turnover in PKU were ambiguous, though the 4 studies examining BTM in children and/or adults with PKU found significant alterations in one or more marker. Investigated markers were heterogeneous and populations studied were not similar in age and thus cannot be reliably compared. Examining correlations between Phe concentration and individual BTM provides mixed results. Differences in findings could be due to differences in methods to measure and report Phe and diversity in reported markers. Consensus on the clinical utility of BTM including reliable methods of measurement and reference ranges, and the establishment of markers suitable for (various age groups of) patients with PKU must be established in future investigations.

### Other factors related to bone status in PKU

Other indicators of bone status that were investigated in patients with PKU were BMC, vitamin D, PTH, calcium, phosphorus and magnesium concentrations. Most outcomes were reported by a small number of studies with heterogeneous groups of patients and, sometimes, contradictory outcomes. BMC may be reduced in non-compliant individuals with PKU, but the clinical implications of low BMC are unknown. Vitamin D and PTH status do not seem to influence BMD based on found results. Calcium seems to be lower in children with PKU, but the impact on bone is ambiguous. Phosphorus and magnesium blood levels do not seem to affect bone status. At this time, it is not possible to draw conclusions on these indicators of bone status without additional evidence from high-quality studies in large groups of patients with consistent measurements. Although the results are inconclusive, including additional bone status indicators in future studies could add to the standard evaluation of bone health in patients with PKU of all ages.

### Summary of evidence

We examined the strongest current evidence on bone health in patients with PKU. All studies were of adequate to high quality, with low to no risk of bias and included only patients who were early diagnosed and treated. Our results suggest that patients with PKU have lower BMD as shown by the mean effect sizes in our meta-analyses. Clinical significance of these outcomes is debatable as the mean effect size Z-scores are within the range for normal BMD according to ISCD recommendations. Though prevalence of low BMD for chronological age is higher in patients with PKU than in the normal population (estimated 10% vs 2.3%, assuming a normal distribution), definitions used for osteopenia and osteoporosis are highly heterogeneous between studies and ISCD positions and WHO standards are rarely followed. Even though several studies reporting on limited cohorts of patients report osteopenia and hypothesize poor bone health in patients with PKU, our review and meta-analyses of all available data suggests bone is not clinically compromised in most early treated patients with PKU.

With the data at hand, we do not have sufficient evidence to establish conclusions on BTM and other indicators of bone status we examined, nor define relationships between Phe or nutrient intake and bone. Further research with more consistent measurements in larger studies is necessary to provide better insight.

### Clinical implications

Mean total body, lumbar spine, and femoral hip BMD Z-scores in patients with PKU are lower than in their healthy peers, but well within the reference range for normal. The clinical relevance of a slightly lower BMD Z-score is unclear. A projected 10% of patients have a BMD Z-score below -2; however 90% of early treated patients with PKU are not at risk for low BMD. Fracture risk must be established before developing final conclusions on bone health in patients with PKU.

In order to evaluate the risk for skeletal fragility or fractures in individual patients, a single assessment of BMD by DXA scan in all adolescent patients with PKU could be considered [33]. Patients with a BMD Z-score above -2 may not require additional follow-up; however patients with low BMD for chronological age (Z-score below -2) and/or a significant fracture history may need follow-up.

For prevention and treatment of low BMD, factors related to bone health in healthy individuals may be applied to prevent low BMD in patients with PKU. We suggest following recommendations for the general population outlined in the ‘National Osteoporosis Foundation’s Clinician’s Guide to Prevention and Treatment of Osteoporosis’ to preserve bone strength [53]. In particular, an adequate intake of calcium and vitamin D, lifelong participation in regular weight-bearing and muscle-strengthening exercise, cessation of tobacco and excess alcohol use if applicable, and treatment of risk factors for falling are also appropriate recommendations for patients with PKU.

### Future studies

Forthcoming studies will need to establish whether slightly lower BMD from an early age increases the risk for osteoporosis or fractures acutely or long-term. Furthermore, for patients with low BMD, preventative and treatment strategies to improve BMD in PKU should be defined. To harmonize evidence, where to measure BMD; valid markers of bone turnover; and definitions of osteopenia/osteoporosis, metabolic control, dietary compliance and protein intake must be concretely defined and standardized and related to fracture risk. Finally, studies are needed of factors impacting BMD that may not be related to PKU such as physical activity.

### Strengths and limitations

One strength of this review is the inclusion of only early-diagnosed and treated patients. By excluding studies on patients who were late diagnosed and at-risk for nutrient deficiencies and potentially impairments in physical activity, which are known to have a negative impact on bone status, we excluded significant potential ascertainment bias. Another strength is that two metabolic centers from two continents performing independent searches reached the same conclusion before combining efforts. Finally, we were able to include a large group of patients in our meta-analysis by contacting authors personally to request data, resulting in meta-analyses of Z-scores from multiple BMD sites.

Our study also has some limitations. There were differences in methodology between study team 1 and study team 2 during individual literature searches, quality and risk of bias assessment, and data extraction, though standardized tools were used by each study team. After comparing data extraction, overview tables were compared and essentially identical. Therefore data extraction was easily and justifiably combined.

Neither search included “fractures” as a clinical outcome in search terms; however, search 1 was broad and captured all literature related to bone in PKU including articles related to fractures.

A small number of the included articles report a correlation between BMD and height of the patient. This is a known restriction of BMD measured by DXA [28]; patients with lower height for age may be falsely diagnosed with a low BMD . Based on the data published however, we were not able to provide height adjusted BMD Z-scores for our pooled data.

Finally, all included studies reported osteopenia and osteoporosis based on Z-scores, contrary to ISCD positions which do not recommend these diagnoses in pediatric patients. Instead, the ISCD recommends the term “low BMD for chronological age” when Z-scores are less than or equal to -2, and does not recommend the diagnosis of osteoporosis without a clinically significant fracture history [28]. These are important caveats to the current literature in patients with PKU and important evidence that criteria for low BMD, osteopenia, and osteoporosis must be concretely defined.

## Conclusions

BMD in early diagnosed and treated patients with PKU is below healthy population average but within normal range. These findings are important to provide preliminary evidence that bone does not appear to be compromised to the extent previously hypothesized. However, while the overall effect size of BMD Z-scores are between -0.4 and -1 in patients with PKU, there is lack of data on a corresponding higher risk of fracture in these patients.

Other indicators of bone status in early treated patients with PKU are inconclusive due to the small number of studies and the heterogeneity in groups examined and measurement methods. Though we now conclude that low BMD does not seem to be an exaggerated concern in patients with PKU, research is needed on the effect of the PKU diet on bone, the reliability of bone turnover markers in bone assessment, and a concrete estimate of fracture risk in patients with PKU.

## Additional file

Additional file 1: Table S1. Articles included after study selection in both search 1 and 2 (n = 13).

## Abbreviations

AND: The academy of nutrition and dietetics
AND EA Process: The academy of nutrition and dietetics evidence analysis process
BMC: Bone mineral content
BMD: Bone mineral density
BTM: Bone turnover markers
CDC: Centers for disease control and prevention
DXA: Dual-energy X-ray absorptiometry
FBMD: Femoral bone mineral density
ISCD: International society for clinical densitometry
LBM: Lean body mass
LBMD: Lumbar bone mineral density
NHANES: National health and nutrition examination survey
Phe: Phenylalanine
PKU: Phenylketonuria
pQUS: Peripheral quantitative ultrasound
PRISMA: Preferred reporting items for systematic reviews and meta-analyses
PROSPERO: International prospective register of systematic reviews
PTH: Parathyroid hormone
QCC: Quality criteria checklist
SDs: Standard deviations
SIGN: Scottish intercollegiate guidelines network
TBMD: Total bone mineral density
WHO: World health organization
