# Supplementary material for: Bone health in phenylketonuria: a systematic review and meta-analysis
Source: Orphanet J Rare Dis. 2015 Feb 15;10:17. doi: 10.1186/s13023-015-0232-y (PMC4340652; doi:10.1186/s13023-015-0232-y)
Supplement: Additional file 1: Table S1. — Articles included after study selection in both search 1 and 2 (n = 13). [file 13023_2015_232_MOESM1_ESM.doc]

Additional file 1: Table S1: Articles included after study selection in both search 1 and 2 (n=13)

| **Reference** | **Study aim** | **In- and exclusion criteria** | **Study type** | **Study population** | **Outcome measures** |
| --- | --- | --- | --- | --- | --- |
| **Coakley KE, Douglas TD, Singh RH. Using predictive modeling to estimate bone mineral density in children and adults with phenylketonuria. Journal of Inherited Metabolic Disease. 2013;36(2):S126. Reference [46]** | To use clinical parameters collected in PKU patients to predict total body BMD. | Included:  Patients age 4 years and older, early treated PKU patients.  No exclusion criteria. | Cohort study  (conference abstract) | N = 57  ♂ 34  ♀24  Mean age  17.3 ± 11 y | - BMD measured by DXA: Z-score TBMD |
| **De Groot MJ, Hoeksma M, Van Rijn M, Slart RHJA, Van Spronsen FJ. Relationships between lumbar bone mineral density and biochemical parameters in phenylketonuria patients. Molecular Genetics and Metabolism. 2012;105(4):566-570.**  **Reference [14]** | To investigate the relationships between  Z-scores of lumbar BMD and age, clinical severity of PKU, mean  Phe concentration and Phe variation of the year prior to DXA scanning,  as well as blood concentrations of vitamins, minerals, and alkaline  phosphatase. | Included:  Patients with PKU diagnosed by newborn screening, early and continuously treated, free of concomitant disease.  No exclusion criteria. | Cohort study  (full article) | N = 53  ♂ 25  ♀ 28  Median age 16 y  Age range  2 – 35 y | - BMD measured by DXA: Z-score LBMD  - blood vitamin D, alkaline phosphatase |
| **Mendes AB, Martins FF, Cruz WMS, Da Silva LE, Abadesso CBM, Boaventura GT. Bone development in children and adolescents with PKU. Journal of Inherited Metabolic Disease. 2012;35(3):425-430.**  **Reference [20]** | To describe the impact of dietary factors and control of plasma Phe levels on bone age and BMD. | Included:  Patients with PKU diagnosed by newborn screening.  No exclusion criteria. | Cohort study  (full article) | N = 13  ♂ 4  ♀ 9  Age range  8-16 y | - BMD measured by DXA: Z-score LBMD  - X-ray of hand and fist: bone age |
| **Adamczyk P, Morawiec-Knysak A, Pludowski P, Banaszak B, Karpe J, Pluskiewicz W. Bone metabolism and the muscle-bone relationship in children, adolescents and young adults with phenylketonuria. Journal of Bone and Mineral Metabolism. 2011;29(2):236-244. Reference [13]** | To asses bone metabolism in young subjects with PKU using routine DXA parameters, functional muscle-bone analysis estimated on the basis of DXA measurements and several laboratory variables. | Included:  Patients with PKU diagnosed by newborn screening who started treatment within the first month of life.  No exclusion criteria. | Cohort study  (full article) | N = 45  ♂ 25  ♀20  Mean age  13.8 ±5.2 y | - Bone turnover markers: ICTP, bALP, osteocalcin  - BMD measured by DXA: Z-score LBMD and TBMD  - BMC  - Other: parathormone, calcitonin, total and ionized calcium |
| **Nagasaka H, Tsukahara H, Takatani T, et al. Cross-sectional study of bone metabolism with nutrition in adult classical phenylketonuric patients diagnosed by neonatal screening. Journal of Bone and Mineral Metabolism. 2011;29(6):737-743. Reference [21]** | To obtain fundamental data for establishing an optimal treatment strategy for bone disease in PKU. | Included:  Adult patients with PKU diagnosed by newborn screening and who were early treated.  No exclusion criteria. | Cohort study  (full article) | Patients  N = 34  ♂ 13  ♀ 21  Age range  20 - 35 y  Controls  N = 36  ♂ 14  ♀ 22  Age range  19 - 40 y | - Bone turnover markers: bALP, OC, procollagen type 1 carboxyterminal propeptide (PICP),  - Other:  vitamin D, PTH |
| **Lage S, Bueno M, Andrade F, et al. Fatty acid profile in patients with phenylketonuria and its relationship with bone mineral density. Journal of Inherited Metabolic Disease. 2010:1-9.**  **Reference [15]** | To assess the influence of fatty acid profile on BMD in patients with PKU. | Included:  genetically proven PKU, no neurological damage and independently functioning in daily activities.  No exclusion criteria. | Cohort study  (full article) | Patients  N = 47  ♂ 30  ♀ 17  Age range  6 – 42 y  Controls  N = 77  ♂ 65  ♀ 12  Age range  6 -45 y | - BMD measured by DXA: Z-score LBMD and FBMD  - Other:  Fatty acid profile, phenylalanine, calcium, 25-hydroxy vitamin D, dietary control and calcium intake |
| **Porta F, Roato I, Mussa A, et al. Increased spontaneous osteoclastogenesis from peripheral blood mononuclear cells in phenylketonuria. Journal of Inherited Metabolic Disease. 2008:1-4.**  **[Reference 48]** | To investigate spontaneous osteoclastogenesis in patients with PKU compared with that in healthy controls. | Included:  Patients with PKU diagnosed by newborn screening who started treatment within the first month of life.  Excluded:  Patients with suboptimal nutrition, short stature, history of immobility, drugs influencing bone and/or concomitant disease with bone or blood involvement. | Cohort study  (full article) | Patients  N = 20  ♂ 8  ♀ 12  Mean age  14 ± 7.1 y  Controls  N = 20  ‘Age- and sex-matched’ | - Osteoclast number and size |
| **Modan-Moses D, Vered I, Schwartz G, et al. Peak bone mass in patients with phenylketonuria. Journal of Inherited Metabolic Disease. 2007;30(2):202-208.**  **Reference [45]** | To evaluate peak bone mass in adult PKU patients and to relate BMD to nutritional parameters. | Included:  Patients with classical PKU diagnosed by newborn screening who started treatment within the first month of life.  No exclusion criteria. | Cohort study  (full article) | N = 31  ♂ 13  ♀ 18  Mean age  25 ± 5.3 y | - BMD measured by DXA: Z-score LBMD and FBMD  - Other: vitamin D, PTH, alkaline phosphatase, calcium |
| **Barat P, Barthe N, Redonnet-Vernhet I, Parrot F. The impact of the control of serum phenylalanine levels on osteopenia in patients with phenylketonuria. European Journal of Pediatrics. 2002;161(12):687-688.**  **Reference [41]** | To determine the role of the control of phenylalanine blood levels in the pathogenesis of osteopenia. | Included:  Patients with PKU diagnosed by newborn screening who were early and continuously treated.  No exclusion criteria. | Case-Control study  (full article) | N = 13  ♂ 7  ♀ 5  Mean age  not described, study concerning pediatric patients | - BMD measured by DXA: Z-score LBMD |
| **Zeman J, Bayer M, Stepan J. Bone mineral density in patients with phenylketonuria. Acta Paediatrica, International Journal of Paediatrics. 1999;88(12):1348-1351.**  **Reference [16]** | To asses total body BMD and lumbar BMD in patients with PKU in relation to nutritional intake and proteins from natural foods and artificial sources. | Included:  Patients with classical PKU diagnosed by newborn screening who started treatment within the first month of life.  No exclusion criteria. | Cohort study  (full article) | N = 44  ♂ 19  ♀ 25  Mean age  16.1 y  Age range  6 -29 y | - BMD measured by DXA: Z-score TBMD and LBMD |
| **Schwahn B, Mokov E, Scheidhauer K, Lettgen B, Schonau E. Decreased trabecular bone mineral density in patients with phenylketonuria measured by peripheral quantitative computed tomography. Acta Paediatrica, International Journal of Paediatrics. 1998;87(1):61-63.**  **Reference [12]** | To measure BMD in patients with PKU using peripheral QCT to determine if osteopenia occurs. | Included:  Patients with PKU diagnosed by newborn screening, treated according to accepted recommendations.  No exclusion criteria. | Cohort study  (full article) | Patients  N = 14  ♂ 8  ♀ 6  Age range  5-28 y  Controls  N = 14  ♂ 8  ♀ 6  Age range 5-28 y | - BMD measured by peripheral QCT: total bone BMD and spongy bone BMD of the radius  - plasma Phe. |
| **Hillman L, Schlotzhauer C, Lee D, et al. Decreased bone mineralization in children with phenylketonuria under treatment. Eur J Pediatr. Jul 1996;155 Suppl 1:S148-152.**  **Reference [37]** | To assess the effect of diet and/or disease on BMD in patients with PKU by comparing children with PKU to non-PKU children for parameters of bone mineralization and homeostasis. | Included:  Pediatric patients with PKU.  No exclusion criteria. | Cohort study  (full article) | Patients  N = 11  ♂ 5  ♀ 6  Mean age  10.9 ±4.2 y  Controls  N = 64  ♂ 32  ♀ 32  Mean age  11.4 ±4.2 y | - Bone turnover markers: Carboxyterminal telopeptide of type I collagen (ICTP), (bALP), osteocalcin in serum. Tartrate resistant acid phosphatase, calcium/creatinine ratio in urine.  - BMD measured by DXA: Z-score LBMD and FBMD  - Other: calcium, magnesium, zinc, phosphorus, albumin, creatinine,  25-hydroxy vitamin D, 1.25 dihydroxy vitamin D, PTH |
| **Allen JR, Humphries IRJ, Waters DL, et al. Decreased bone mineral density in children with phenylketonuria. American Journal of Clinical Nutrition. 1994;59(2):419-422.**  **Reference [17]** | To evaluate TBMD and SBMD and nutrient intake in a group of children with PKU on dietary therapy in comparison with a group of age-matched control subjects | Included:  Pre-pubertal patients with PKU diagnosed by newborn screening, early and continuously treated. | Cohort study  (full article) | Patients  N = 32  ♂ 20  ♀12  Mean age  7.7 ± 2.3 y  Controls  N = 95  ♂ 57  ♀ 38  Mean age   - 1. ± 2.1 y | - BMD measured by DXA: Z-score LBMD and TBMD |

♂ included male subjects, ♀ included female subjects.
